# A case of accidental intraperitoneal placement of the rectus sheath block catheter via an out-of-plane approach

**DOI:** 10.1186/s40981-024-00705-4

**Published:** 2024-04-03

**Authors:** Ai Ono, Keisuke Yoshida, Rieko Oishi, Satoki Inoue

**Affiliations:** Department of Anesthesiology, Fukushima Medical University School of Medicine, 1, Hikariga-Oka, Fukushima, Fukushima 960-1295 Japan

To the editor,

Continuous nerve blocks have become widely used for postoperative analgesia in recent years. Ultrasound-guided catheter insertion is commonly performed using an in-plane or out-of-plane approach [1]. However, it is unclear which approach is more effective. Herein, we report a case in which a catheter for continuous rectus sheath block unexpectedly reached the abdominal cavity, via an out-of-plane approach.

A 67-year-old woman underwent bilateral rectus sheath blocks with a catheter-through-needle technique (Hakko Disposable Pain Clinic Set®, Hakko, Japan) after open surgical repair of abdominal aortic aneurysm. On ultrasound, due to the surgical wound, the posterior sheath of the rectus abdominis muscle could not be visualized well in the long-axis view with a linear probe; thus, we placed two catheters (left and right sides) under the short-axis view of the rectus sheath muscle using an out-of-plane approach, while injecting a small amount of drug solution to confirm the tips of needle. When we advanced the needle to the proper location, we observed the spread of local anesthetic (0.25% levobupivacaine, 20 mL per one side) along the rectal sheath. Then, we inserted the catheter, rigidly holding the needle in place so it would not shift from its position. However, the tip of the catheter was not clearly visible on ultrasound, and we did not evaluate the spread of local anesthetics administered through the catheter. On the Postoperative Day 1, routine postoperative computed tomography revealed that the right catheter had strayed into the abdominal cavity (Fig. 1). This catheter was quickly removed, resulting in no complications.

**Fig. 1:**
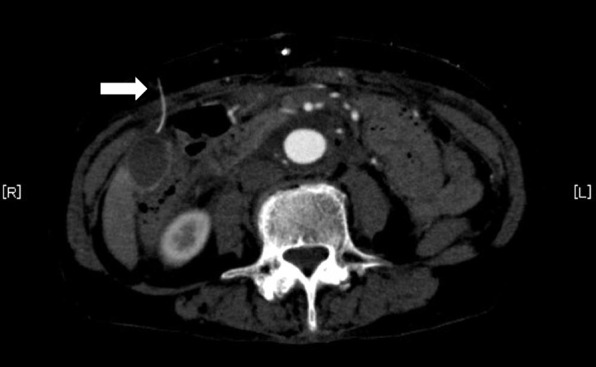
A computed tomography scan (transverse view) on Postoperative Day 1. The white arrow in the figure indicates the catheter, which strayed into the abdominal cavity

The primary advantage of the in-plane approach is its safety, because the entire needle can be visualized during the procedure. The primary disadvantage is its narrow ultrasound beam width of ≤ 1 mm, which makes the visibility of the needle difficult [2]. Furthermore, the distance from the skin to the target tends to be longer using this approach, possibly resulting in reduced visibility in deeper areas [3]. In contrast, the out-of-plane approach has the advantage of good maneuverability of the needle compared to the in-plane approach [2]. However, it has the drawback of not visualizing the entire needle; that is, only a point of the needle is visible, but that point is not necessarily the “true” needle tip [4]. Additionally, the needle we used for the patient in the current report was a non- echogenic needle; thus, the needle tip was difficult to identify. Consequently, this drawback of the out-of-plane approach with this type of needle may have led to the outcome of the present case. In addition, on reflection, we also suspect that inadequate evaluation of the catheter tip position (after the needle was removed) contributed to both the outcome and the delay in detection. If the needle or the target is not clearly visible under ultrasound, the nerve block procedure must be discontinued, and alternative analgesic methods should be used.

In conclusion, we believe that we should be aware of the advantages and disadvantages of the out-of-plane and in-plane approaches when performing ultrasound-guided catheter insertion for continuous nerve block, and nerve block should not be performed if the location of the needle/catheter cannot be definitively confirmed by ultrasound.

## Data Availability

Not applicable.
